# Supervised Physiotherapy Improves Three-Dimensional (3D) Gait Parameters in Patients after Surgical Suturing of the Achilles Tendon Using an Open Method (SSATOM)

**DOI:** 10.3390/jcm11123335

**Published:** 2022-06-10

**Authors:** Andrzej Czamara, Łukasz Sikorski

**Affiliations:** 1 Department of Physiotherapy, The College of Physiotherapy in Wrocław, 50-038 Wrocław, Poland; sikorski.lukasz.92@gmail.com; 2 Center of Rehabilitation and Medical Education in Wrocław, 50-038 Wrocław, Poland

**Keywords:** three-dimensional, kinematics, spatiotemporal analysis, walking, ankle joint, Achilles tendon, rehabilitation

## Abstract

Background: The aim of this study was to assess the effectiveness of 38 supervised postoperative physiotherapy (SVPh) visits conducted between 1 and 20 weeks after SSATOM on the values of 3D gait parameters measured at 10 and 20 weeks after surgery. Material: Group I comprised male patients (*n* = 22) after SSATOM (SVPh x = 38 visits) and Group II comprised male patients (*n* = 22) from the control group. Methods: A non-randomized, open-label, controlled clinical trial was performed in the two groups to obtain the following values: Step length (cm), stride length (cm), step width (cm), next stance phase (%), swing phase (%), double support (%), gait velocity (m/s), and walking frequency (step/min). The measurements were carried out using the BTS SMART system (Italy). Results: Orthopedic examination showed no pain, a negative result of Thompson and Matles tests, and proper healing of Achilles tendon (ultrasound image). In Group I, between 10 and 20 weeks after SSATOM, there was a statistically significant improvement in all tested gait parameter values (*p* ≤ 0.001 to 0.009). Conclusions: Conducting 38 SVPh visits significantly improved the values of the analyzed kinematic and spatiotemporal gait parameters in patients in the twentieth week after SSATOM, which were mostly close to the non-operated side and the results of the control group. However, the gait speed and stride length were not close to the results of the control group.

## 1. Introduction

The contraction of human triceps muscles of the Achilles tendon (AT) during locomotion, generates plantar flexion and propulsion of the foot [1]. In recent years, the number of AT lesions has been increasing, reaching up to 70 cases per 100,000 people in the population [2]. The problem often concerns middle-aged and young men who are physically active [3]. In the case of AT rupture, the orthopedist conducts a thorough history and clinical test as well as pain assessment of the patient [4]. An ultrasound examination of AT is considered as a standard procedure in these cases, and if necessary, additional examinations are performed [5,6]. Achilles tendon injuries are treated surgically or conservatively [7,8]. A review of the literature indicates the predominance of surgical treatment over the conservative treatment [7,8,9,10]. The goal of postoperative physiotherapy is to gradually restore the function of the tendon, ankle joint, the entire limb, and then independent walking. Subsequent stages of supervised postoperative physiotherapy (SVPh) should restore the patients’ optimal physical activity [11]. At present, there are as yet, no unequivocal answers to the following question: What is the impact of postoperative physiotherapy on the behavior of kinematic and spatiotemporal gait parameters using an open method (SSATOM), which is conducted and supervised by a physiotherapist in patients after surgical suturing of the Achilles tendon [12,13,14].

In the literature, only a few publications were found, that meet the requirements of a standardized description of conducted and supervised physiotherapy after SSATOM [11,15,16,17,18,19,20]. Moreover, some interesting studies concerning the discussed research problem were conducted on a small number of patients after AT injury [13,21]. According to Tengman et al. (2013), 2 to 5 years after the surgical treatment of AT, patients had signs of tendon elongation, as well as low muscle strength and function, especially during walking [22]. Sun et al. (2020) conducted a study of gait analysis in patients 2 years following the surgical suturing of AT. The authors found that a restraint of plantar flexion of the foot along with a greater deficit in muscle strength is responsible for plantar flexion of the foot on the operated side, resulting in the increased risk in knee injury during gait [23]. Jadacka et al. (2017) [24] had similar conclusions as Sun et al. (2020) [23].

Heikkinen et al. (2017) showed that the non-surgical treatment of AT rupture resulted in significantly greater soleus muscle atrophy and AT elongation compared with the surgical treatment. Patients treated surgically had on average 10 to 80% greater calf muscle strength compared with patients treated conservatively [25]. Speedtsberg et al. (2019) observed that even 4.5 years after the non-operative treatment of the injured AT, patients had impaired peak dorsiflexion values, reduced total strength of the concentric and plantar flexor, as well as decreased quasi-stiffness in the initial and final range of dorsal flexion of the foot, especially during walking [26]. Aufwerber et al. (2021) showed that the more accelerated rehabilitation regimen with early functional mobilization did not lead to a more symmetrical gait pattern neither at 8 weeks nor at 6 months after SSATOM [12].

Lins et al. (2013) noted that 12 months after AT surgery a crucial improvement was observed in the values of walking speed, walking frequency, stride length, and percentage share of the support phase compared with the control group. Finally, the authors found few gait disturbances during walking compared with 6 months after surgery [21]. Nordenholm et al. (2022) showed that performing a surgery after the AT rupture and postoperative rehabilitation may be an effective way to improve the values of the biomechanical parameters of walking after the incident [27]. However, even 12 months after surgery, patients showed significantly lower values of gait speed, step width, shorter stride length, longer relative stance phase, and power disturbances during walking compared with the control group [27]. Chan et al. (2011) conducted an analysis of gait in two groups of patients after the surgical suturing of the AT, using the open and the mini-open methods. At the twelve month after surgery, no significant differences were found between the study groups in the time of support and swing, and the length of a single step [28]. Don et al. (2007) evaluated the clinical condition of muscle strength and gait analysis in patients who had undergone surgical suturing of a complete AT rupture at 3, 6, 12, and 24 months. At 24 months, a deficit in calf-muscle eccentric strength was present, determining adaptive changes in gait strategy [14]. Alvit et al. (2017) showed that the double support on the non-operated limbs and the support time on the operated limbs were significantly lower in the sixth month after the surgery compared with the control group [29].

The authors of this study assumed that in order to improve the kinematic and spatiotemporal parameters of gait in patients after SSATOM, early and careful postoperative physiotherapy, should be initiated based on a specific protocol of and SVPh conducted by a physiotherapist in consultation with the treating physician [11]. Subsequently, the duration, number, and frequency of SVPh visits with the patient should be taken into account. Subsequently, the duration, number and frequency of SVPh visits with the patient should be taken into account, as well as the patient’s exercises performed at home [11,16,30,31].

The aim of the study was to evaluate the effectiveness of 38 supervised postoperative physiotherapy visits, that were performed between the first and twentieth week after surgical suturing of the Achilles tendon, on the 3D gait parameters measured at 10 and 20 weeks after surgery.

## 2. Materials and Methods

The research was conducted at the Center of Rehabilitation and Medical Education and the College of Physiotherapy in Wrocław, in accordance with the guidelines of the Declaration of Helsinki. The participants and the Ethics Committee at the College of Physiotherapy in Wrocław No. 1/2019, the Ethics Committee at the College of Physiotherapy in Wrocław No. 1/2012, and the Senate Committee of The Ethics of Research at the Academy of Physical Education in Wrocław 2006 (18 May 2006) provided written consent for the research. The study was designed as an open-label, non-randomized, controlled clinical trial and was conducted between 2010 and 2019. Short title: “Evaluation of the Effectiveness of Physiotherapy on 3D gait after Achilles Tendon Surgery”.

### 2.1. Participants

**Figure 1:**
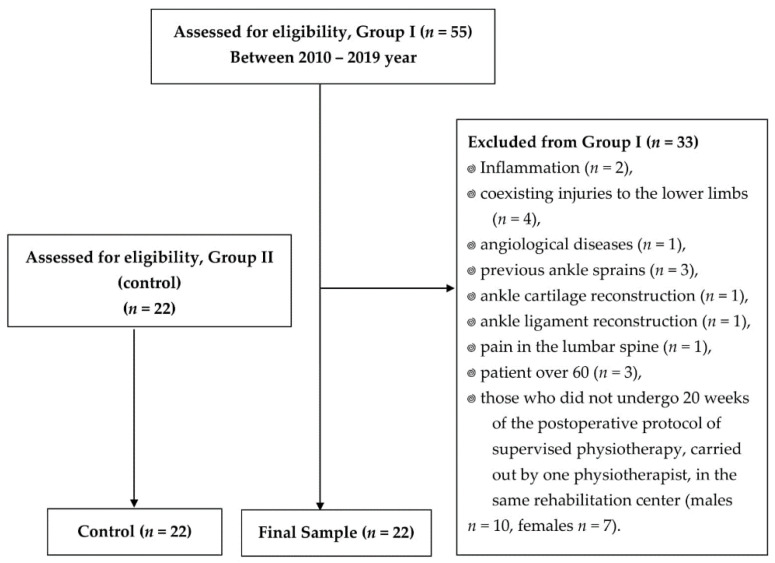
Flow diagram of the study; *n*–number of individuals.

Initially, 55 patients (*n* = 48 men and *n* = 7 women) were included in the study, after surgical suturing of the Achilles tendon using an open method (SSATOM). The following inclusion criteria were applied: Unilateral surgical suturing of Achilles tendon (AT) using the open method, conducting postoperative supervised physiotherapy in patients by one physiotherapist, in accordance with one physiotherapy protocol, absence of concomitant lesions of the lower limbs, spine and concomitant diseases, and individuals between 20 and 60 years of age. Physiotherapy was carried out in the same rehabilitation center, using the same devices and experimental apparatus. On the basis of history and medical records, some patients were excluded from the initial group due to the following reasons: Inflammation (*n* = 2), coexisting injuries to the lower limbs (*n* = 4), angiologic diseases (*n* = 1), previous ankle sprains (*n* = 3), ankle cartilage reconstruction (*n* = 1), and ankle ligament reconstruction (*n* = 1). Subsequently, patients with pain in the lumbar spine (*n* = 1), patients who were more than 60 years old (*n* = 3) and less than 20 years old (*n* = 0), as well as those who did not undergo 20 weeks of the postoperative protocol of supervised physiotherapy, carried out by one physiotherapist, in the same rehabilitation center (*n* = 10 men and *n* = 7 women) (Figure 1) were excluded from the study [11]. Finally, 22 men with no AT rupture or other concomitant lower limb injuries were qualified for Group II (Figure 1).

Furthermore, in Group I, the men (*n* = 22) after SSATOM, who had an operation on 10 right lower limbs and 12 left lower limbs, were qualified for further analysis. Patients in Group I had 21 right dominant lower limbs and one left dominant lower limb. The men in Group II of the control group, with no AT injuries (*n* = 22), had 20 right dominant lower limbs and 2 left dominant limbs. In Group I, patients after a unilateral complete rupture of the AT had an open method of surgical AT suturing using Keesler’s approach and immediately after surgery, they had anticoagulant treatment, and analgesics [32]. The characteristics of the dominant lower limbs, including those operated on in Group I, and the dominant limbs in Group II are presented in Table 1. Both groups were not significantly different in age, body height and weight.

### 2.2. Supervised Postoperative Physiotherapy (SVPh)

General assumptions of the physiotherapeutic procedure: When planning, SVPh visits had to be performed between the first to twentieth week after surgical suturing of Achilles tendon using an open method (SSATOM) in the outpatient Rehabilitation Center, instituted three visits per week, conducted by an experienced physiotherapist, during the subsequent stages of this therapy, for patients who, after the AT surgery, planned to return to their daily activities and successively to recreational physical activity. Each SVPh visit lasted for 2 h at the Rehabilitation Center. For professional athletes, five SVPh visits per week were accepted. Supervised postoperative physiotherapy was carried out based on the protocol described above [11]. This section presented a shorter scheme of the physiotherapeutic protocol, as reported in Table 2 [11]. In addition, each patient was instructed by a physiotherapist on how to perform the therapeutic exercises at home, depending on the stage of postoperative physiotherapy. In Group I, the patients completed an average of 37.82 SVPh visits (it was hypothesized that they completed 38 SVPh visits). During the first 10 weeks after SSATOM, the patients completed an average of 14 SVPh visits (individually from 1 to 32 visits) (Table 1). The average time to start SVPh after SSATOM was 5.14 ± 2.14 weeks (the earliest individual visit was from the first week after SSATOM). The mean frequency of SVPh visits per week was 1.36 between 1–10 weeks after SSATOM, and 1.89 between 1–20 weeks after SSATOM (Table 1). The physiotherapist did not have an influence on the regularity of patients’ participation in this therapy, who ultimately made their own decisions about the number and regularity of the visits. Additional factors that could influence the process of SVPh in the first 10 weeks are as follows: Excessive pain and swelling, problems with postoperative scar, the patients’ motivation to treatment, varied level of physical fitness before Achilles tendon injury, compliance with the specific recommendations made by a physician, conducting the exercises recommended by the physiotherapist at home or non-adherence to the recommended amount and intensity of exercises to be performed individually. The exclusion criteria we adopted excluded patients who had excessive pain and inflammation (Figure 1). However, 10% of patients pursuing SVPh had periodic swelling and pain, which, however, resolved in the course of the treatment. The factors described above resulted in a periodic reduction in the frequency and total number of visits.

**Table 2:** Characteristics of the five-stage supervised postoperative physiotherapy (SVPh) program for patients after surgical suturing of Achilles tendon (SSATOM).

Stage of SVPh	Weeks after SSATOM	Short Protocol of SVPh [11]
**I**	**Up to 2 weeks after the surgery**	**Exercises for shank and foot muscles:** - isometric exercises of shank and foot muscles in brace, - foot proprioception exercises on the ball in closed kinematic chains in orthosis on the surface of the wall in the supine position, with the elevation of the lower limb, - re-education of gait with the two elbow crutches in ankle brace. In addition, the physiotherapist instructed the patient on how to perform the exercises at home and cool the Achilles tendon area every 6 h.
		**Exercises for other parts of the body:** - strengthen the muscles of the upper limbs and shoulder girdles, - isometric exercises of a large group of lower limb muscles.
		**Physical therapy treatment:** - cooling the operated area of Achilles tendon using Cryo/Cuff for 15 min. After a few days, applying the cryotherapy treatment for 2–3 min. After a stiumlation exercise with a variable low-energy magnetic field, laser stimulation of the postoperative scar.
**II**	**3 to 4 weeks after the surgery**	The physiotherapeutic procedure from the previous stage was continued and additionally: **Exercises for shank and foot muscles:** - active exercises of short foot muscles on the surface of the wall in the supine position, without straining the operated ankle joint, - the passive movement (CPM) of the plantar was continued, and to a limited extent, the dorsal flexion of the foot, as well (see Figure 2), - re-education of gait with the two elbow crutches in ankle orthosis, - active exercises of short foot muscles on the surface of the floor in a sitting position on a chair, without straining the ankle joint, - partial load of the operated lower limb on the base of the strain gauge platform with the foot immobilized in an orthopedic shoe. Prior to starting this exercise, a measurement of the vertical component of ground reaction forces (vGRF) in Newtons (N) is carried out using MTD balance platforms, while standing on one leg (see Figure 3a,b) and then on two legs (see Figure 4a,b). The patient’s task was to stand on a non-operated limb on one platform. Then, with an operated limb (in immobilization of the ankle joint with an orthopedic shoe), the patient had to load the other platform without the occurrence of pain and to maintain a balance. Next, the person that carried out the research asked the patient to stand still in this position for 15 s, not to hold the handrail, and on the command of “start” began to record the vGRF values (see Figure 4a). In Figure 4b, the lower record demonstrates the vGRF values of the operated lower limb and the upper record represents the vGRF values of the non-operated lower limb. The measurement time was 15 s. On the basis of the results obtained from the measurement presented in Figure 4b, for the operated lower limb, we individually selected the value of 180 N and to this value, the patient may partially load the substratum of the platform (see Figure 5a). The method of performing this exercise is as follows: The patient stands on the non-operated limb on one leg on one platform for about 6 s, and while securing himself on the platform railings with his hands, partially loads the operated limb for 6 s on the other platform, not exceeding the value of 180 N (see Figure 5a,b). This type of exercise is repeated individually by the patient for 2 to 3 min in three series. The break between sets is 1 min. The exercise was terminated when pain occurred. The load was gradually increased every 3–4 days, ranging from 30 to 50 N, provided that there was no pain or swelling in the area of the Achilles tendon of the operated leg. Additionally, the physiotherapist instructed the patient on how to perform the exercises at home and cool the Achilles tendon area every 6 h.
		**Exercises for other parts of the body:** - active exercises with partial resistance of the muscles, such as quadriceps, iliopsoas, buttock, and rotating hip joints and trunk in positions that did not cause an overload of the operated ankle joint.
		**Physical therapy treatment:** - electrodiagnostic and electrostimulation of the triceps surae muscles alternated the anterior groups’ muscles acting on the ankle joint, - phonophoresis with non-steroidal, anti-inflammatory drugs or heparin on the Achilles tendon and ankle area, in the case of persistent pain or swelling.
		**Manual therapy:** - therapeutic dry massage of Achilles tendon area, foot, ankle joint and shank massage, special techniques, e.g., Kibler, lymphatic drainage of the entire lower limb (under the condition that the wounds are healed after surgery), - first-degree micromobilization of Achilles tendon, hindfoot, forefoot, and gastrocnemius.
	**5 to 6 weeks after the surgery**	The physiotherapeutic procedure from the previous stage was continued and additionally: **Exercises for shank and foot muscles:** - active plantar and dorsal flexion of the foot, - passive motion (CPM), successively active-passive pronation and supination of the foot, - cycle ergometer without load, with ankle joint immobilized by a brace, - progressive pressure of the foot on the ground with a load on the operated lower limb during exercises on platforms, e.g., within 6 weeks after SSATOM (see Figure 6), Additionally, the physiotherapist instructed the patient on how to perform the exercises at home.
**III**	**7 to 12 weeks after the surgery**	The physiotherapeutic procedure from the previous stage was continued and additionally: **Exercises for shank and foot muscles:** Progressive pressure of the foot on the ground, with a load on the operated lower limb during exercises on platforms up to 80% of the bodyweight value. Successively, the load was gradually increased up to 100% of bodyweight values (without an orthopedic shoe) between 8 and 9 weeks after surgery. For example, if the patient’s bodyweight value is 67 kg, then when standing on one leg on the platform the value of the reaction force of the vertical component of the platform of about 670 N (Figure 7) is triggered (the word concerning results from the unstable equilibrium state in humans). It should be hypothesized that a value of 670 N corresponds to a bodyweight of 67 kg (1.0 bodyweight). Successively, the load was gradually increased every 3–4 days with a value of 0.1 bodyweight without a shoe, - re-education of the gait performance of individual phases. Initially, with partial relief of the lower limb with crutches without the brace, then without crutches, provided that the patient achieved 100% of the load on the operated limb (value of 1.0 bodyweight), - improving the gait performance of individual phases after reaching the value of the ground reaction forces of the vertical component of about 1.2 bodyweight. The final decision was made by the orthopedist after an orthopedic examination and ultrasound, - isometric with partial resistance, without causing pain to the muscles that are responsible for plantar and dorsal flexion of the foot on the operated side as well as for setting the foot angle outside of the excessive stretching zone of the operated Achilles tendon. Initially, the resistance was 30%, then 40% of the value of the maximum isometric tension. Successively, the resistance was increased by about 10% every week, provided that there was no pain: - active short muscles of the foot with the partial load on the ankle joint, - active plantar and dorsal flexion, pronation and supination of the foot, - stretching muscles, such as short foot muscle, triceps surae muscle, soleus muscle, - proprioception in closed kinematic chains on trampolines and other devices with unstable ground, - Progressive pressure of the foot on the ground, with a load on the operated lower limb during exercises on platforms. Progressive pressure of the foot on the ground, with a load on the operated lower limb during exercises on platforms 1.3 to 1.4 of the bodyweight value (see Figure 8a,b) about 12 weeks after SSATOM. Successively, the load was gradually increased every 3–4 days by 0.1 of bodyweight values without a shoe, - improving the technique of performing individual phases of walking without crutches on a treadmill (in the following weeks, the walking speed was increased, the angle of inclination of the treadmill was increased, the walking distance was gradually extended, provided that there was no pain), - re-education of independent ascent and descent, initially on low steps of stairs, - heel raise exercise on both legs.
		**E** **xercises for other parts of the body:** - large muscle groups of the upper and lower limbs, the torso of the pelvic girdle, with stabilization of the whole body, - a bicycle ergometer without an orthosis (time from 10 to 15 min, frequency of 60–70 revolutions per minute). Initially, without load, next, the load was started from 25 W for 5 min. Without interrupting, the intensity was maintained (increased by 5 W every 2 min). In the following weeks, this type of exercise was started from 40–50 W, and after 6 min, the load was increased by 5–10 W every 2 min, - stretching muscles, such as hamstrings, iliopsoas muscle, quadriceps muscle, and fascia lata, - improving neuromuscular coordination.
		**Physical therapy treatment:** Electrodiagnostic and electrostimulation of the triceps surae muscles of the injured leg.
		**Manual therapy of injured tissues:** - therapeutic dry massage, transverse massage, point massage, special techniques, e.g., Kibler, massage in the aquatic environment every 3 days, - mobilizations (first degree and successively after 2–3 weeks second-degree mobilization was introduced), Achilles tendon, hindfoot, forefoot, gastrocnemius muscle, and foot short muscles.
		**Additional comments:** The physiotherapist instructed the patient on how to perform the exercises at home.
	**13 to 16 weeks after the surgery**	The physiotherapeutic procedure from the previous stage was continued and additionally: **Exercises for shank and foot muscles:** - submaximal strength of the muscles acting on the ankle joint and the entire operated and non-operated limbs, - Progressive pressure of the foot on the ground, with a load on the operated lower limb during exercises on platforms to around 1.5 of the bodyweight value. Successively, the load was gradually increased every 4–5 days by 0.1 to 0.2 of bodyweight values in the following weeks, provided there was no pain or swelling, - isometric, eccentric, and concentric–eccentric exercises with partial progressive resistance, - eccentric and concentric muscles of the foot and lower legs with bodyweight resistance, - re-education of the gait technique of individual phases, walking on stairs, walking on toes, - dynamic prioprioception.
		**E** **xercises for other parts of the body:** - improving the advanced level of neuromuscular coordination, - restoring a slow jog at the end of this stage of physiotherapy.
**IV**	**17 to 24 weeks after SSATOM**	The physiotherapeutic procedure from the previous stage was continued and additionally: **Exercises for shank and foot muscles:** - heel raise exercise on the injured legs, - the dynamic strength of the muscles acting on the ankle joints, - exercises that were aimed at helping the patient return to physical work or sports that required a physical activity of the Achilles tendon and ankle joint.
		**Exercises for other parts of body:** - squats, - the dynamic strength of the muscles acting on the muscles of the lower limbs, - running, - interval running, - gradually running quickly, - vertical and countermovement jumping exercises, - plyometric exercises.
		**Additional comments:** The physiotherapeutic procedure from the previous stage was continued.
**V**	**24 to 28 weeks after SSATOM**	**Exercises for shank and foot muscles:** The physiotherapist performed and supervised the patient’s exercises including running with maximum speed, changing movement directions, and specific exercises aimed at improving power, speed, and agility, adapted for a particular sport and the individual patient’s capabilities. **Additional comments:** - the physiotherapist instructed the patient on how to perform the exercises at home

### 2.3. Orthopedic Examination

Concerning the subjects in Groups I and II, prior to the registration of the values of kinematic and spatiotemporal gait parameters, a specialist orthopedic physician, conducted a medical examination, which included the patient’s history, pain assessment using a visual analogue scale (VAS), and AT efficiency values with the Matles and Thompson tests. Subsequently, the doctor assessed the ankle joint structure, its stability, and continuity of AT through palpation and ultrasound [33]. Whereas, the subjects in Group I, prior to proceeding with the following stages of postoperative physiotherapy and before the last registration and measurement of the kinematic and spatiotemporal gait parameters in the twentieth week after SSATOM, had another orthopedic examination carried out in accordance with the schemes mentioned above. All of the subjects in Groups I and II obtained regular orthopedic results and were admitted to the next stage of the study.

### 2.4. Three-Dimensional Gait Analysis Registration of Kinematic and Spatiotemporal Parameter Values

In Group I, the registration and subsequent evaluation of the kinematic and spatiotemporal gait parameters on hard ground were carried out at 10 and 20 weeks after surgical suturing of AT, using the optoelectronic three-dimensional recording system (BTS Bioengineering, Milan, Italy, 2006) [34]. Six infrared camcorders (with a recording frequency of 120 Hz) and two piezoelectric Kistler platforms (with a recording frequency of 960 Hz) were used. Calibration was performed prior to each test, in accordance with the manufacturer’s instructions [34]. Anthropometric measurements were carried out, using a diameter compass for the measurement of both lower limbs, pelvis, such as pelvic width (cm) measured from the anterior superior iliac spine, pelvic depth (cm) measured from the anterior superior iliac spine to the greater trochanter of the femur on both sides of the body, width of the knee joints (cm) measured between the condyles of the femur, width of the ankle joints (cm) measured from the lateral to the medial malleolus, and the relative length of the lower limbs (cm). Additionally, the height and bodyweight of the subject were measured. The data obtained were entered into the computer system (BTS Bioengineering, Milan, Italy, 2006), in accordance with the manufacturer’s instructions [34]. The subject was wearing a swimsuit, and after disinfection of the skin, 22 markers were glued on both lower limbs, starting from the fifth head of the metatarsal bone, on the lateral malleolus, the posterior loftiness of the calcaneus, the middle part of the fibula, the head of the fibula, the lateral condyle of the femur, half the length of the femur, in the place of the greater trochanter, the anterior superior iliac spines, and at the level of S1 vertebra. Moreover, three markers were glued in the upper body at the height of the C7 vertebra and on the right and left shoulder appendage [35]. The measurement started by standing on both legs on a strain gauge platform for 10 s with the upper limbs positioned along the trunk. In the further part of the measurement, the participants were asked to walk 7 m along a path starting with each step from the left-lower limb, after hearing the Go command. Six measurements of walking along the path in the gait laboratory were performed, and each of them was carried out under identical conditions. After the test, the report was prepared using BTS capture, tracker, and analyzer programs [33,36]. For further analysis, two measurements were selected using the programs mentioned above, which contain 100% of the gait cycle of the first and last heels in contact with each lower limb [37]. Additionally, during the gait analysis, the patient had to perform a contact of each foot with one of the two Kistler platforms. The following values were measured: Step length (cm); stride length (cm); step width (cm), stance phase (%), swing phase (%), double support (%), gait velocity (m/s), and walking frequency (step/min) using the six-phase gait cycle [37]. In the control subjects of Group II, measurements of the kinematic and spatiotemporal gait parameters were performed using the same method in Group I, but were carried out only once.

### 2.5. Test of the Covered Distance When Walking on Unstable Treadmill Ground

To assess the ability to cover the walking distance on unstable ground, the subjects in Groups I and II had to walk 4 km on a treadmill at a speed of 6 km/h (1.66 m/s). The speed of 6 km/h corresponded to the fast pace of walking by the men in both groups, taking into account their body height, weight, and age, as presented in Table 1. The patients in Group I performed this test at week 20 after surgical suturing of Achilles tendon. The condition for passing this test was to cover a distance of 4 km without pain and swelling on the day after the end of the test, as well as on the following day. Covering the distance of 4 km of marching without rest on unstable ground allowed the patients to take 7000 to 8000 steps (single step from 50 to 60 cm), which are recommended by the American Heart Association to ensure adequate health-promoting activities for adults [38].

### 2.6. Statistical Analysis

For statistical analysis, Microsoft Office Excel 2007 (Microsoft, Redmond, WA, USA) and IBM SPSS 23 (IBM, Armonk, NY, USA) were used. We started our analysis with the calculation of mean values (M) and standard deviations (SD). The Shapiro–Wilk test was used to verify the normality of the distribution of the tested variables. Independent tests were used for the comparison of age, body height, and weight as well as the between-group values. For intra-group comparisons, tests for dependent groups were used. Repeated-measures ANOVA with Tukey’s HSD as post-hoc test determined the intra-group and inter-group values of spatiotemporal and kinematic gait parameters in Group I in 10 and 20 weeks after surgical suturing of Achilles tendon using an open method. One-way ANOVA was used for the comparison of the values obtained for the right operated limb in Group I and the left limb in Group II including step length (cm), stride length (cm), step width (cm), stance phase (%), swing phase (%), double support (%), and gait velocity (m/s). When the significance level was at *p* < 0.05, Tukey’s post-hoc test was performed for a group with the same number of people. Pearson’s linear correlation coefficient (r) was calculated for the strength and direction of the linear relationship between the number of visits to the supervised physiotherapy and the results of kinematic and spatiotemporal gait parameters. The values corresponding to all of the two-dimensional associations were classified as negligible (0.00–0.30), low (0.31–0.50), moderate (0.51–0.70), high (0.71–0.90), and very high (0.901–1.00) [39].

## 3. Results

### 3.1. Evaluation of Kinematic and Spatiotemporal Parameter Values Using a Three-Dimensional Gait Analysis (3D) System

In Group I, at the tenth week of postoperative supervised physiotherapy after surgical suturing of Achilles tendon using an open method (SSATOM), statistically significant disturbances in values of step length, stance phase, and swing phase were noted between the involved and uninvolved lower limbs (*p* < 0.001). In addition, 10 weeks after SSATOM in Group I, the values of step length, stride length, gait velocity, and walking frequency values, were statistically significantly lower in involved and uninvolved legs compared with the study conducted at the twentieth week after surgery, respectively for the involved and uninvolved legs (*p* < 0.001). Then, between the tenth and twentieth weeks after SSATOM in Group I, there was a statistically significant improvement in weeks for all of the analyzed biomechanical gait parameters (from *p* ≤ 0.009 to *p* ≤ 0.001), as shown in Table 3. Comparison of the particular week of each of the examined lower limbs (weeks x limb) obtained statistical significant improved parameters in 20 weeks after SSATOM of step length, stance phase, swing phase, and double support (Table 3).

On the basis of analysis of variance, statistically significantly lower values of stride length, swing phase, and gait velocity of the operated limbs were shown in Group I at 20 weeks after SSATOM compared with the right and left limbs of Group II (from *p* ≤ 0.001 to *p* = 0.014, Table 4). Tukey’s test revealed statistically significantly lower values of stride length, swing phase, and gait velocity of the operated limb in Group I compared with the right and left limbs in Group II (from *p* ≤ 0.001 to *p* = 0.039, Table 4). In all of the presented values of gait parameters, no statistically significant differences were found between the right and left limbs in Group II (Table 4).

### 3.2. Test of the Covered Distance When Walking on Unstable Treadmill Ground

In Group I, all of the patients after SSATOM walked a distance of 4 km on a treadmill at a speed of 1.66 m/s (6 km/h), without pain (VAS = 0) and the occurrence of AT swelling after the test and during the next day. In Group II, the patients walked a distance of 4 km on a treadmill at a speed of 1.66 m/s (6 km/h), without pain and the appearance of AT swelling after the test and during the next day.

### 3.3. Last Orthopedic Examination Results

The latest results of the orthopedic examination preceded the last recording and measurement of the kinematic and spatiotemporal gait parameters, as well as the gait test on a treadmill over a distance of 4 km in Group I in the twentieth week after SSATOM. In Group II (control), the study was conducted once. The results showed no pain on the VAS scale, negative Thompson and Matles test results in both study groups.

### 3.4. Association of Number of Supervised Postoperative Physiotherapy Visits with Kinematic and Spatiotemporal Parameter Values

A significantly positive and moderate correlation was noted between the higher number of visits during the first to the tenth week of supervised postoperative physiotherapy and higher gait velocity values for operated and non-operated limbs, respectively (r = 0.581; *p* = 0.005 and r = 0.542; *p* = 0.009) and stride length (r = 0.544; *p* = 0.009 and r = 0.544; *p* = 0.009), as presented in Table 5 A significantly negative and low correlation was noted between the higher number of visits up to 10 weeks after SSATOM of postoperative physiotherapy and lower step width values for operated and non-operated limbs (r = −0.475; *p* = 0.025, r = −0.475; *p* = −0.475; *p* = 0.025) and for the stance phase of the non-operated limb (r = −0.430; *p* = 0.046). A significantly positive and moderate correlation was found between the greater number of supervised postoperative physiotherapy (SVPh) visits in the period from the first to the tenth week for the swing phase of the non-operated limb (r = 0.503; *p* = 0.017), as shown in Table 5.

## 4. Discussion

For the purpose of this study, the authors have shown that conducting 38 supervised postoperative physiotherapy visits between 1 and 20 weeks after surgical suturing of the Achilles tendon resulted in significant improvements in the analyzed kinematic and spatiotemporal gait parameters obtained at week 20 compared with the results obtained 10 weeks after surgery. In addition, at 20 weeks after surgery, these gait parameters were mostly similar to the parameters obtained on the non-operated side and to the results of the control group without Achilles tendon injury. However, the gait velocity, stride length and, to a lesser extent, the swing phase of the operated limbs were lower than the results obtained in the control group.

In this study, the authors aimed to capture the interest of other researchers to the problem of retainment of 3D gait parameters, while conducting the complex treatment that included surgery after a complete AT rupture, immobilization, postoperative pharmacotherapy, and successively postoperative physiotherapy that is conducted and supervised by a physiotherapist. In particular, the authors paid attention to the latter element of treatment. Therefore, a detailed protocol and the total number of supervised postoperative physiotherapy (SVPh) visits, weekly frequency, duration of physiotherapy, precise procedures of applied treatment, and exercises for each 2-h physiotherapy visit were described above. Moreover, for each stage of physiotherapy, the authors specified recommendations for the home-based exercises that should be performed by the patients.

The literature analysis indicates that some of the authors presented a biomechanical assessment of gait in patients treated conservatively [22,26,40,41,42] and surgically [21,27,43] after AT rupture. Moreover, studies indicate that the authors used various methods and surgical techniques [44,45,46]. Different physiotherapy protocols and the level of accuracy of the description of these protocols, as well as the duration of physiotherapy, are known [11,16,28]. Some publications do not contain a description of physiotherapeutic procedures or are limited to providing basic schemas of early postoperative management [15,22,43].

In reference to the results obtained by other authors, who assessed the behavior of the biomechanical gait parameters over a period of 6 to 12 months, as well as in a longer period of time after AT surgery [12,13,14,21,27,28,29], our study showed a significant improvement in the twentieth week after surgical suturing of Achilles tendon using an open method (SSATOM) in most of the analyzed values of 3D gait parameters. However, the authors of the current study did not achieve the restoration of all gait parameters in patients after SSATOM, which would be similar to the non-operated side and the results of the control group. In addition, other studies showed a correlation between the higher number of SVPh visits in the first 10 weeks after SSATOM, which may indicate a favorable behavior of the analyzed biomechanical gait parameters between the operated and non-operated lower limb. However, the evaluation of the results obtained on the basis of correlation is not a sufficient tool and should be extended in the future to include an analysis, e.g., linear regression.

Several factors may affect the obtained results of the study of the complex treatment of patients after SSATOM, including the result of the performed surgery, the type and time of immobilization after the operation, pharmacological support, and individual patients’ predisposition for tissues’ healing of AT after surgery. Other conditions include adaptation to postoperative recommendations, age, gender, patients’ motivation for the conducted treatment or possible concomitant diseases. The last stage of complex treatment after acute rupture of AT is postoperative physiotherapy. In this study, the authors hypothesized that the average frequency of postoperative SVPh would be three visits per week with a physiotherapist. However, these proposed assumptions were not fully implemented by the patients. In addition, the authors’ recommendations did not have any influence on the systematicity of the patients’ planned participation process in postoperative physiotherapy. Moreover, we did not reach a valid answer to the following question: Is it possible to better improve the values of analyzed 3D gait parameters in Group I patients by conducting an average of three visits per week, in accordance with the planned SVPh protocol, and thus with a higher total number of SVPh visits in the 20 weeks after SSATOM? On the other hand, other studies have shown that only time after treatment has a full influence on the return of symmetrical and normative 3D gait parameters of patients after Achilles tendon rupture [12,13,14,21,27,28,29].

The presented research entails some limitations. Initially, our research showed that individually, patients differed in the timing of the early initiation of individual physiotherapy that is conducted and supervised by a physiotherapist. Moreover, the analysis showed that our patients had different regularities and frequencies of conducted and supervised postoperative physiotherapy after SSATOM. Despite our recommendations, we had no influence on the number and frequency of these visits due to the individually varying regularity of patients’ participation in postoperative physiotherapy. In the future, this problem must be resolved. The solution to the presented problem will probably allow us to finally determine whether a higher number and frequency of conducted physiotherapy, and its early but also safe implementation after surgery SVPh, will have an impact on the dynamics and improvement of the 3D biomechanical gait parameters in patients after SSATOM. The current studies should be treated as an early evaluation and these studies were intended to be used for the ongoing monitoring of changes in gait biomechanics in relation to the clinical evaluation in the current physiotherapeutic treatment. Furthermore, in the future, it would be necessary to continue the presented research at a distant date after the AT surgery. Then, prior to the registration and measurement of 3D gait parameter values, the patient should be advised to cover the walking distance as quickly but as safely as possible, and not only to walk the distance “at his own pace”.

The reason for obtaining a lower walking speed during the measurement of the values of 3D biomechanical gait parameters on hard ground in patients after SSATOM, compared with a much higher walking speed on a treadmill, was the adopted study methodology, not the lack of ability to walk faster.

As well known, it is difficult to compare gait results on stable and unstable grounds. On the other hand, the authors wanted to point out that the gait assessment should be carried out in both of the presented ground conditions and should also take into account the supplementation of the 3D gait assessment with practical tests similar to the patients’ daily needs. In addition, covering the distance of 4 km of marching without rest on unstable ground allowed the patients to take 7000 to 8000 steps, which are recommended by the American Heart Association to ensure health-promoting activities for adults [38].

In the future, the retainment of the values of the 3D gait parameters presented during this treatment should be compared with other surgical techniques [47], possible complications, and ways of preventing these complications [48]. Another limitation is the lack of participation of women in the current research. Seven female patients were excluded from the study as they did not meet the hypothesized 20 weeks of SVPh. In addition, 10 male patients were excluded from further studies for the same reason. Silbernagel et al. (2015) suggested that the small number of women with Achilles tendon injuries is due to the fact that they have better blood supply conditions of AT and are less physically active than men [49].

We would like to emphasize that the description and subsequent step-by-step implementation of SVPh visits, in accordance with the therapeutic protocol described above, is as important as describing in detail the surgical technique, immobilization and selection, and dosage of the recommended drugs. This comprehensive treatment approach may allow for better standardization of treatment in the future. Moreover, the standardization of the SVPh protocol will allow for a real comparison of the results of our research, using the presented physiotherapy protocol, especially in terms of the number of visits, and their frequency, for different groups of patients (athletes, professional athletes, people practising sports for recreational purposes, and the elderly). Furthermore, the standardization of the last stage of treatment, which is postoperative physiotherapy in patients after complete rupture to the AT, would allow for a comparison of the results obtained in our research with the results obtained by other authors.

Nevertheless, conducting an open-label, non-randomized, controlled clinical trial research can be susceptible to bias. Therefore, this type of study should be carried out as a randomized clinical trial to increase the value of the research. Furthermore, it would be necessary to continue the research with more participants in the study groups.

## 5. Conclusions

Our research demonstrated that conducting 38 supervised physiotherapy visits in patients between 1 and 20 weeks after surgical suturing of the Achilles tendon, resulted in significant improvements of all the analyzed kinematic and spatiotemporal gait parameters at week 20 compared with the results obtained 10 weeks after surgery. In addition, at 20 weeks after surgery, these gait parameters were mostly similar to the non-operated side and to the results of the control group without Achilles tendon injury.

However, the gait velocity, stride length and, to a lesser extent, the swing phase of the operated limbs were lower than the results obtained in the control group.

## Figures and Tables

**Figure 2:**
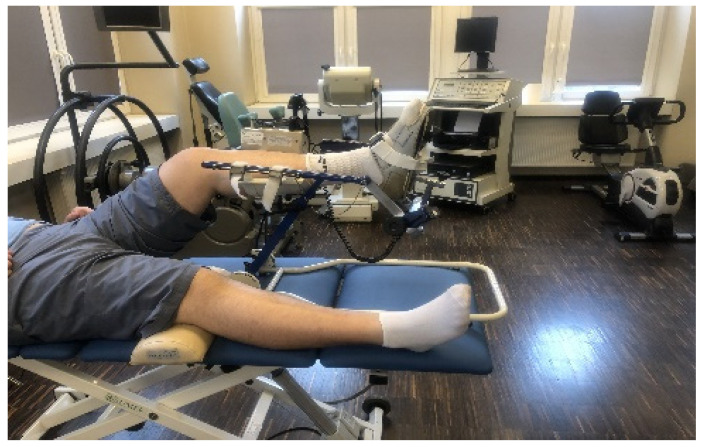
Exercise of continuing passive movements of plantar flexion of the ankle joint on the operated side, with limitation of dorsal flexion of the foot after SSATOM.

**Figure 3:**
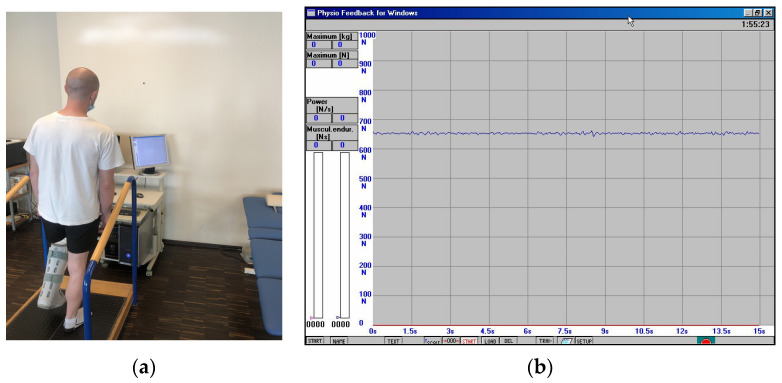
(**a**) Single-leg standing test on non-injured leg; (**b**) the individual measurement of the ground reaction force values for the vertical component (vGRF) measured in Newtons (N), blue line—non-injured leg.

**Figure 4:**
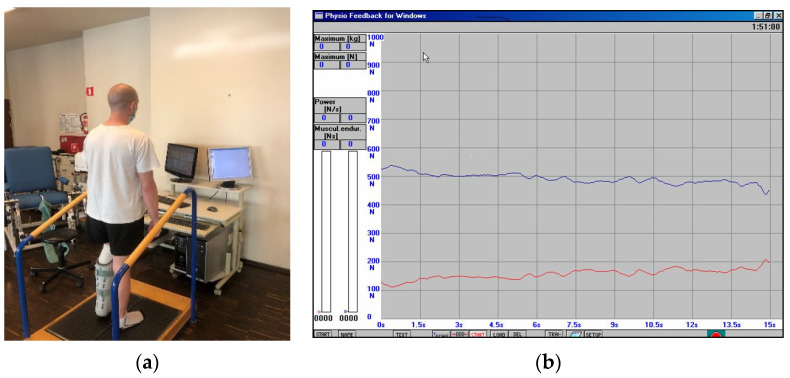
(**a**) The individual measurement of the ground reaction force values for the vertical component (vGRF) measured in Newtons (N) in a lax free-state on both legs; (**b**) the measurement of both legs (red line—operated leg, blue line—non-injured leg).

**Figure 5:**
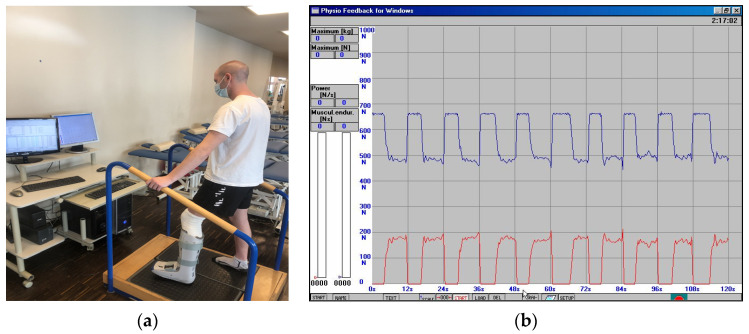
(**a**,**b**) Record of the report from one session of exercises individually selected with a value of 180 N, to which the patient could partially load the platform substrate with a limb operated for vGRF values, 3 weeks after SSATOM. This value was determined in the baseline measurement (Figure 3b) (red line—operated leg, blue line—non-injured leg).

**Figure 6:**
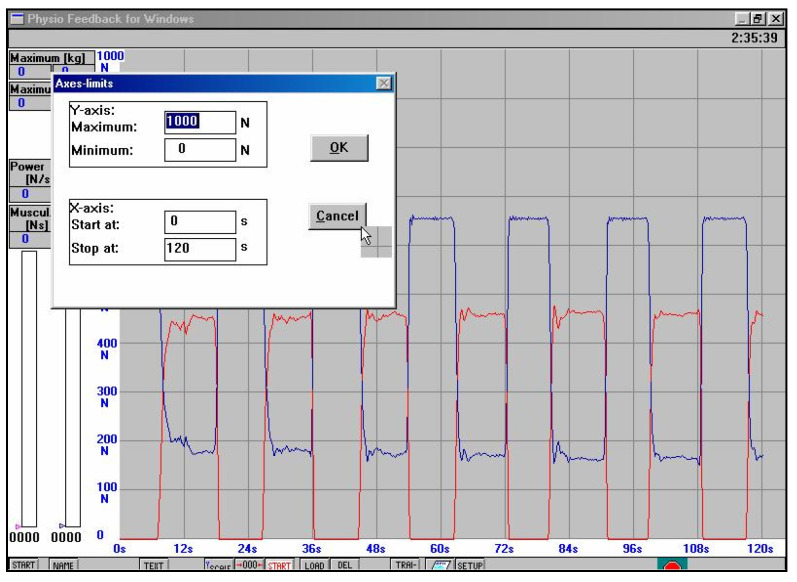
Record of the report from one session of exercises individually, to which the patient could partially load the platform substrate with a limb operated for vGRF values, 6 weeks after SSATOM, (red line—operated leg, blue line—non-injured leg).

**Figure 7:**
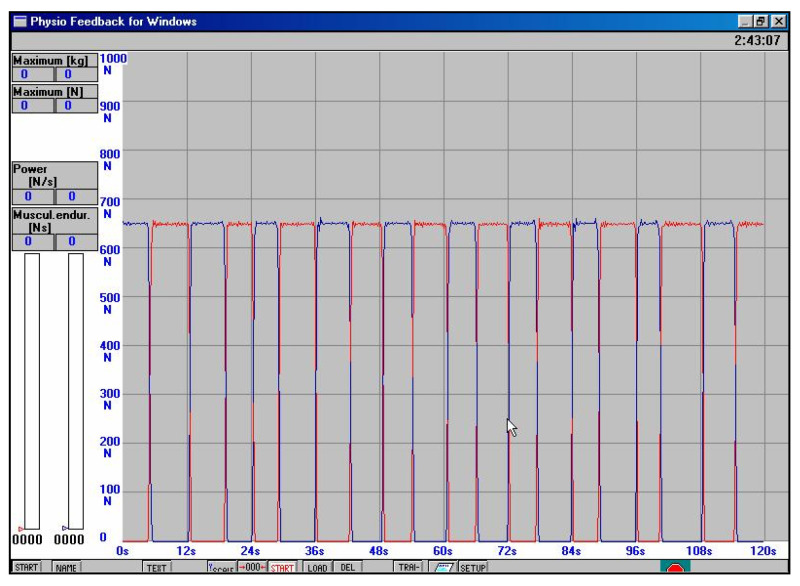
The individual result obtained by the patient for the value of the ground reaction forces (N) for the vertical component, while standing on one leg, and alternately, on the operated and non-operated lower limb. After conversion to kilograms, this corresponds approximately to the patient’s bodyweight during the exercise sessions on a strain gauge platform, within 8 weeks of physical therapy after surgical suturing of Achilles tendon, (red line—operated leg, blue line—non-injured leg).

**Figure 8:**
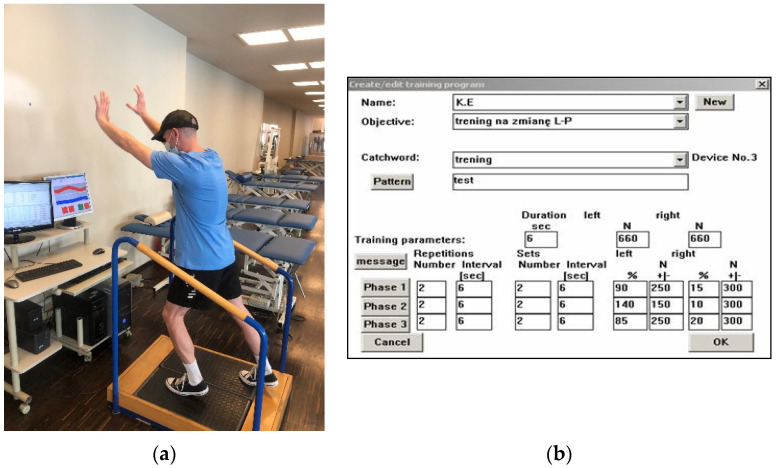
(**a**,**b**) Exercise with progressive load on the platform substrate with a lower limb operated for vGRF values, up to 1.4 bodyweight values.

**Table 1:** The between-group comparison of the values corresponding to age, body mass, body height, the duration of postoperative physiotherapy, the number of postoperative physiotherapy sessions presented between 1–10 weeks and 1–20 weeks after surgical suturing of Achilles tendon, time to start of supervised physiotherapy after surgical suturing of Achilles tendon, average weekly frequency visits of postoperative physiotherapy sessions presented between 1–10 weeks and 1–20 weeks after surgical suturing of Achilles tendon, dominant limb, and operated limb between Group I and Group II (control).

	Group I (*n* = 22) (M ± SD)	Group II (Control) (*n* = 22) (M ± SD)	*p*-Value
Age (years)	36.82 ± 7.4	36.68 ± 6.14	0.947 ^†^
Body weight (kg)	88.32 ± 10.04	86.27 ± 13.22	0.566 ^†^
Body height (cm)	183.18 ± 8.4	181.36 ± 6.4	0.424 ^†^
Dominated limb	P = 21; L = 1	P = 20; L = 2	-
Injured limb	P = 10; L = 12	P = 0; L = 0	-
Duration of SVPh (weeks)	20	n/a	-
Number of SVPh sessions from 1 to 10 weeks after SSATOM	13.64 ± 9.39 (individually from 1 to 32 visits)	n/a	-
Number of SVPh sessions from 1 to 20 weeks after SSATOM	37.82 ± 16.79 (individually from 13 to 77 visits)	n/a	-
Mean time to start SVPh after SSATOM	5.14 ± 2.14 (individually from 1 to 8 weeks after SSATOM)	n/a	--
Average weekly frequency of SVPh visits from 1 to 10 weeks after SSATOM	1.36 ± 0.94 (visits per week)	n/a	-
Average weekly frequency of SVPh visits from 1 to 20 weeks after SSATOM	1.89 ± 0.84 (visits per week)	n/a	-

Mean values (M); standard deviations (SD); statistical significance level (*p*); the number of individuals (*n*); not applicable (n/a); supervised postoperative physiotherapy (SVPh); surgical suturing of Achilles tendon using an open method (SSATOM); ^†^ *t*-test.

**Table 3:** Comparison of the intra-group and inter-group values of spatiotemporal and kinematic gait parameters in Group I in 10 and 20 weeks after SSATOM.

Parameters	Limb/Leg	Weeks		*p*-Value ^†^ Weeks	*p*-Value ^†^ Limb	*p*-Value ^†^ Weeks × Limb
		10 Weeks after SSATOM (M ± SD)	20 Weeks after SSATOM (M ± SD)			
Step Length (cm)	Involved	41.95 ± 11.98 ^a,b^	60.5 ± 7.89	***p* ≤ 0.001**	***p* ≤ 0.001**	***p = 0.014***
	Uninvolved	25.50 ± 14.73 ^b^	55.95 ± 8.4			
Stride Length (cm)	Involved	75.27 ± 28.89 ^b^	129.14 ± 13.82	***p* ≤ 0.001**	***p = 0.96***	***p = 0.87***
	Uninvolved	76.32 ± 28.30 ^b^	128.55 ± 14.13			
Step Width (cm)	Involved	21.77 ± 3.99	19.73 ± 3.12	***p* = 0.009**	***p = 1.00***	***p = 1.00***
	Uninvolved	21.77 ± 3.99	19.73 ± 3.12			
Stance Phase (%)	Involved	60.08 ± 5.26	60.43 ± 4.02	***p* ≤ 0.001**	***p* ≤ 0.001**	***p* ≤ 0.001**
	Uninvolved	72.81 ± 8.28 ^a,b^	61.83 ± 1.99			
Swing Phase (%)	Involved	39.73 ± 5.20	38.17 ± 2.89	***p* ≤ 0.001**	***p* ≤ 0.001**	***p* ≤ 0.001**
	Uninvolved	26.02 ± 7.99 ^a,b^	40.65 ± 11.93			
Double Support (%)	Involved	27.55 ± 31.08 ^b^	10.91 ± 1.7	***p* ≤ 0.001**	***p =* 0.38**	***p =* 0.044**
	Uninvolved	19.38 ± 20.50	12.7 ± 3.17			
Gait Velocity (m/s)	Involved	0.54 ± 0.28 ^b^	1.11 ± 0.21	***p* ≤ 0.001**	***p =* 1.00**	***p =* 0.88**
	Uninvolved	0.55 ± 0.28 ^b^	1.1 ± 0.21			
Walking Frequency (step/min)	Both legs	83.31 ± 13.45	102.4 ± 10.06	***p* ≤ 0.001** **^ǂ^**		

Mean values (M); standard deviations (SD); statistical significance level (*p*); the number of individuals (*n*); supervised postoperative physiotherapy (SVPh); surgical suturing of Achilles tendon using an open method (SSATOM), *p* ≤ 0.05 are indicated in bold; ^†^ The comparison was performed using repeated-measures ANOVA with a post-hoc test (Tukey’s HSD test). ^a^—statistically significant differences between results of limbs (Tukey’s HSD test); ^b^—statistically significant differences between results of weeks (Tukey’s HSD test); ǂ *t*-test.

**Table 4:** Inter-group comparison of spatiotemporal and kinematic gait parameters between the involved leg of Group I compared with the right and left leg of Group II, measured at 20 weeks after SSATOM.

	Group I 20 Weeks after SSATOM (M ± SD	Group II (Control) (M ± SD)		*p*-Value ^†^	*p*-Value ^ǂ^
	Involved (IL)	Right (RL)	Left (LL)		
Step Length (cm)	60.5 ± 7.89	58.59 ± 5.84	61.23 ± 7.89	0.467	
Stride Length (cm)	129.14 ± 13.82	140.68 ± 9.86	139.36 ± 9.0	**0.002**	IL: RL—***p*** **= 0.003** IL: LL—***p*** **= 0.009** RL: LL—*p* = 0.918
Step Width (cm)	19.73 ± 3.12	20.86 ± 5.11	20.86 ± 5.11	0.634	
Stance Phase (%)	60.43 ± 4.02	60.13 ± 1.53	60.51 ± 2.81	0.903	
Swing Phase (%)	38.17 ± 2.89	39.8 ± 1.43	39.94 ± 1.88	**0.014**	IL: RL—***p*** **= 0.039** IL: LL—***p*** **= 0.023 ** RL: LL—*p* = 0.976
Double Support (%)	10.91 ± 1.7	10.8 ± 1.63	10.28 ± 1.62	0.401	
Gait Velocity (m/s)	1.11 ± 0.21	1.35 ± 0.14	1.36 ± 0.14	***p* ≤ 0.001**	IL: RL—***p*** **≤** **0.001** IL: LL—***p*** **≤** **0.001** RL: LL—*p* = 0.950

Mean values (M); standard deviations (SD); statistical significance level (*p*); surgical suturing of Achilles tendon using an open method (SSATOM); *p* ≤ 0.05 is indicated in bold; ^†^ one-way ANOVA; ^ǂ^ Tukey test.

**Table 5:** Correlation between the number of postoperative physiotherapy visits conducted between 1–10 weeks, after surgical suturing of Achilles tendon to the obtained spatiotemporal and kinematic gait values in Group I.

Supervised Physiotherapy		Number of SVPh Visits from 1 to 10 Weeks Compared with Values Obtained in 10 Weeks after SSATOM
Stance Phase (%)	Involved	*r* = −0.129; *p* = 0.566
	Uninvolved	***r* = −0.430; *p* = 0.046**
Swing Phase (%)	Involved	*r* = 0.129; *p* = 0.569
	Uninvolved	***r* = 0.503; *p* = 0.017**
Double Support (%)	Involved	*r* = −0.319; *p* = 0.148
	Uninvolved	*r* = −0.207; *p* = 0.355
Step Length (cm)	Involved	*r* = 0.314; *p* = 0.154
	Uninvolved	*r* = 0.344; *p* = 0.117
Gait Velocity (m/s)	Involved	***r* = 0.581; *p* = 0.005**
	Uninvolved	***r* = 0.542; *p* = 0.009**
Stride Length (cm)	Involved	***r* = 0.544; *p* = 0.009**
	Uninvolved	***r* = 0.531; *p* = 0.011**
Step Width (cm)	Involved	***r* = −0.475; *p* = 0.025**
	Uninvolved	***r* = −0.475; *p* = 0.025**
Walking Frequence (step/min)		*r* = 0.382; *p* = 0.08

Surgical suturing of Achilles tendon using an open method (SSATOM); supervised postoperative physiotherapy (SVPh); level of significance (*p*); correlation coefficient (r); *p* ≤ 0.05 are indicated in bold.

## Data Availability

Results are available upon request to the editors and reviewers.
